# Cheek Plumper: An Innovative Anti-cheek Biting Appliance

**DOI:** 10.5005/jp-journals-10005-1352

**Published:** 2016-06-15

**Authors:** Vivek Rana, Nikhil Srivastava, Noopur Kaushik, Prerna Panthri

**Affiliations:** 1 Professor, Department of Pedodontics and Preventive Dentistry Subharti Dental College, Meerut, Uttar Pradesh, India; 2 Principal, Professor and Head, Department of Pedodontics and Preventive Dentistry Subharti Dental College, Meerut, Uttar Pradesh, India; 3 Reader, Department of Pedodontics and Preventive Dentistry Subharti Dental College, Meerut, Uttar Pradesh, India; 4 Postgraduate Student, Department of Pedodontics and Preventive Dentistry Subharti Dental College, Meerut, Uttar Pradesh, India

**Keywords:** Cheek biting, Intraoral cheek biting appliance, Plumper, White flaky keratinization.

## Abstract

One of the most challenging tasks for a pediatric dentist is the management of deleterious oral habits which adversely affect the dentofacial complex. However, if these habits can be intercepted and diagnosed well in time, they can save the patient from the psychological impact of undergoing long treatment therapies. One such rare deleterious oral habit is cheek biting that affects the buccal mucosa. Presented here is a case report which describes the interception of this deleterious habit in a 15-year-old female child who was a bilateral cheek biter with the help of an innovative intraoral appliance: The cheek plumper.

**How to cite this article:** Rana V, Srivastava N, Kaushik N, Panthri P. Cheek Plumper: An Innovative Anti-cheek Biting Appliance. Int J Clin Pediatr Dent 2016;9(2):146-148.

## INTRODUCTION

Oral habits are a major cause for concern for a pediatric dentist. Dorland (1957) defined habits as a fixed or constant practice established by frequent repetition. The prevalence of oral habits in schoolchildren has been quoted to be 29.7% by Shetty and Munshi^2^ in their study. Management of such oral habits is a challenging task for any pediatric dentist and it becomes even more difficult when dealing with a deleterious self-inflicting habit. One such less commonly seen habit is cheek biting, which is a chronic, innocuous, and self-inflicted injury.^3^ It is most commonly seen as a result of psychological stress or an attempt to gain attention.^4^

What makes this even more uncommon is it occurs bilaterally and with grave intensity that it inflicts bleeding from the cheeks. Normally, this is a psychogenic habit which occurs as a result of a wide range of emotions,^5^ there-fore for management of this habit, psychological conditioning of the patient is required along with appliance therapy. The management also becomes more challenging when the habit is deep rooted. Presented here is the case of a 15-year-old girl with this deep-rooted habit of cheek biting and its management with the help of psychological counseling and an innovative removable appliance therapy.

## CASE REPORT

**Fig. 1:**
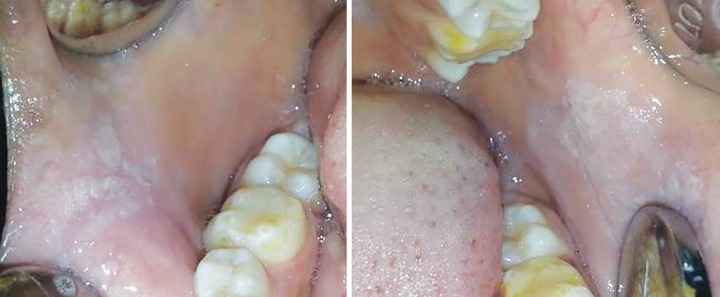
Bilateral white flaky keratinization (preoperative view)

A 15-year-old female child reported to the Department of Pedodontics and Preventive Dentistry, Subharti Dental College, Meerut, with the chief complaint of mild to moderate pain on the inner side of both the cheeks since 1 month. After a set of questions which were asked to the child, she appeared to be very stubborn. Intraoral examination revealed tender diffused white flaky elevations on both sides of the buccal mucosa at the level of the occlusion which were extending anteroposteriorly from the corner of the mouth up to 2nd permanent molar (Fig. 1). The medical history revealed that patient had no systemic illness. Also the personal history of the patient revealed that the patient had the habit of intense cheek biting, often leading to bleeding from the cheeks. Therefore, after counseling the patient, an immediate interception was planned with a unique removable cheek biting appliance.

## FABRICATION OF APPLIANCE

**Fig. 2:**
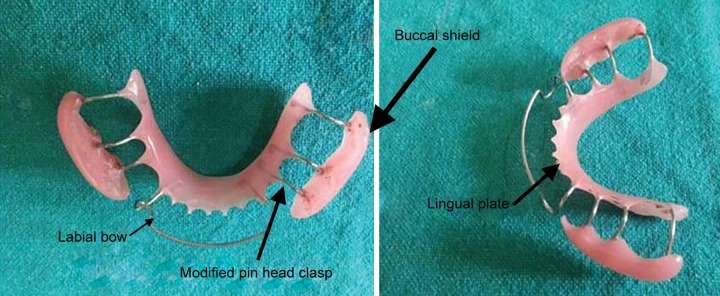
Parts of the fabricated appliance

**Fig. 3:**
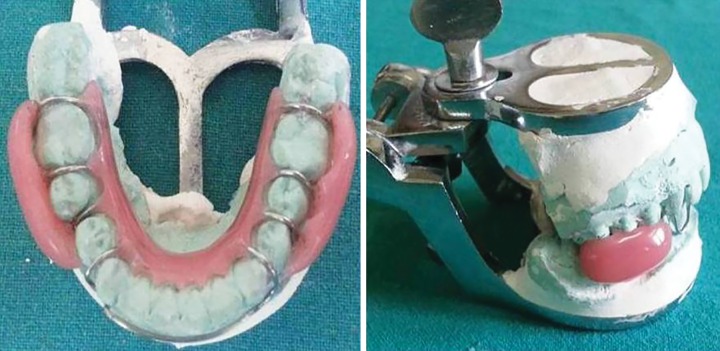
Articulated final trimmed appliance

**Fig. 4:**
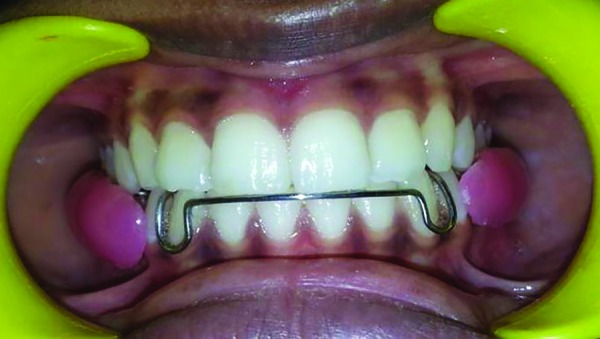
Reflected buccal mucosa with buccal shields

**Figs 5A and B:**
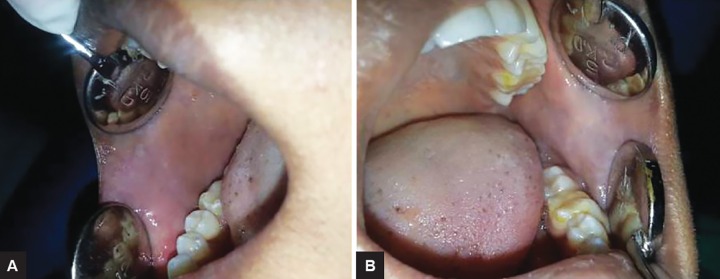
Six months postoperative follow-up

Alginate impressions of maxillary and mandibular arches were recorded and poured in dental stone. Both the casts were then articulated in occlusion. Wire bending was done by using 21 gauge wire on the mandibular cast to incorporate a passive labial bow and modified pin head clasps (Fig. 2). After completion of wire bending, acrylization was done. Two buccal shields extending from premolar to second permanent molar on both sides were made using self-cure acrylic resin which were supported by lingual plate with the aid of modified pin head clasps (Fig. 2). Buccal shields were made in such a way that they had minimal contact with the teeth and soft tissues while from the outer side, the shape was slightly convex in order to keep buccal mucosa away from teeth and promote healing even after the removal of appliance. After acrylization, the appliance was trimmed, polished, and checked for sharp extensions (Fig. 3). This newly trimmed appliance was named as “cheek plumper” because of its primary function to intercept tha habit and protect buccal mucosa from cheek biting injury (Fig. 4). After appliance delivery, postinsertion instructions were given to the patient and was recalled at 1, 3, and 6 months follow-up. After 6 months follow-up, it was observed that the patient had quit the habit and buccal mucosa of both the sides was healed (Figs 5A and B).

## DISCUSSION

Oral habit is defined as an acquired tendency or learned practice of muscular contraction. Morsicatio buccarum or cheek biting habit is less common and is prevalent in 750 out of every one million individuals, with females being more affected than males.^3^ It is mostly deep-rooted as a result of psychological stress or in an attempt to gain attention.^4^ The habit is characterized by marked hyperkeratosis, producing a ragged surface with many surface keratin projections.^5^ Psychological counseling alone does not appear beneficial^6^ as the condition gets worsened due to stress.^7^ Therefore, a removable reminder appliance was fabricated.

For interception of cheek biting habit, various appliances have been advocated like vestibular screen, a mouth guard,^3^ and even a crib.^8,9^ However, none of these was well suited for our case as the child was a young adolescent and the habit was deep-rooted and bilateral in nature. So, keeping in mind considerations, such as patient’s age, general health, ability to cooperate with treatment, and severity of oral injuries, a bilateral cheek biting interception design was planned. Moreover, this design seemed not to interfere with esthetics of the patient. Our unique design effectively deflected the buccal mucosa bilaterally from the occlusal table, thereby preventing trauma. Besides, it is a single unit that can be easily fabricated and installed without discomfort or risk to the patient.

## CONCLUSION

Primary role of a pediatric dentist lies in interception of habit. Thus, the removable prosthesis described in this case presents a conservative treatment approach in managing habitual biting of oral mucosa. It also fulfills all criteria, such as esthetic appearance and a single unit functioning bilaterally without hampering speech. Thereby, this appliance offers an effective way of intercepting one of the often overlooked deleterious cheek biting habits.
